# Work, eat and sleep: towards a healthy ageing at work program offshore

**DOI:** 10.1186/s12889-016-2807-5

**Published:** 2016-02-09

**Authors:** Vanessa Riethmeister, Sandra Brouwer, Jac van der Klink, Ute Bültmann

**Affiliations:** Department of Health Sciences, Community and Occupational Medicine, University of Groningen, University Medical Center Groningen, Antonius Deusinglaan 1, 9713 AV Groningen, The Netherlands

**Keywords:** Offshore, Healthy ageing, Occupational health, Workplace, Needs assessment

## Abstract

**Background:**

Health management tools need to be developed to foster healthy ageing at work and sustain employability of ageing work-forces. The objectives of this study were to 1) perform a needs assessment to identify the needs of offshore workers in the Dutch Continental Shelf with regard to healthy ageing at work and 2) to define suitable program objectives for a future healthy ageing at work program in the offshore working population.

**Methods:**

A mixed methods design was used applying an intervention mapping procedure. Qualitative data were gathered in *N* = 19 semi-structured interviews and six focus-group sessions (*N* = 49). Qualitative data were used to develop a questionnaire, which was administered among *N* = 450 offshore workers. Subgroup analyses were performed to investigate age-related differences relating to health status and work-related factors.

**Results:**

The importance of good working environments, food, as well as sleep/fatigue management was identified by the qualitative data analysis. A total of 260 offshore workers completed the questionnaire. Significant differences in work ability were found between offshore workers aged <45 and 45–54 years (mean 8.63 vs. 8.19; *p* = 0.005) and offshore workers aged <45 and >55 years (mean 8.63 vs. 8.22; *p* = 0.028). Offshore workers had a high BMI (M = 27.06, SD = 3.67), with 46 % classified as overweight (BMI 25–30) and 21 % classified as obese (BMI >30). A significant difference in BMI was found between offshore workers aged <45 and ≥55 years (mean 26.3 vs. 28.6; p <0.001). In total, 73 % of offshore workers reported prolonged fatigue. A significant difference in fatigue scores was found between offshore workers aged <45 and ≥55 years (mean 36.0 vs. 37.6; *p* = 0.024). Further, a “dip” was reported by 41 % of offshore workers. Dips were mainly experienced at day 10 or 11 (60 %), with 45 % experiencing the dip both as physical and mental fatigue, whereas 39 % experienced the dip as only mental fatigue.

**Conclusions:**

Both qualitative and quantitative analyses identified work, food and sleep/fatigue management as most important program objectives for a healthy ageing at work and sustainable employability program offshore. Future studies should investigate possible causes of dip occurrences and high fatigue scores to identify suitable interventions.

**Electronic supplementary material:**

The online version of this article (doi:10.1186/s12889-016-2807-5) contains supplementary material, which is available to authorized users.

## Background

Work-forces are ageing [1, 2]. In view of the expected shortages in workforce, societies and companies have to develop health management policies and practices to foster healthy ageing at work (HA@W) and to sustain employability. Sustaining employability is especially challenging for workers in offshore oil and gas production because of strenuous work conditions (Table 1). Health problems associated with long-term offshore work are likely to increase with age resulting in increases in sickness absences and early retirement claims [2]. Moreover, the physiological and psychological effects of ageing can affect the preparedness to emergency response tasks and thus compromise health and safety offshore [3]. Consequently, there is a need to develop workplace programs with a focus on HA@W to: foster employee health and safety, sustain employability of offshore workers and ensure knowledge transfer and economic means. Developing HA@W programs may help to promote health and reduce the consequences of unhealthy ageing, e.g. work productivity loss, sickness absence and work disability. Recently, Dutch researchers estimated an increase of the financial burden associated with unhealthy ageing from 155 billion Euro in 2010 to 419 billion Euro in 2050 [4]. To foster HA@W and to sustain employability offshore it is important to identify program objectives relevant for HA@W among offshore workers. To our knowledge, intervention studies addressing HA@W offshore have not yet been conducted. Moreover, only a few offshore studies, conducted e.g. in Norway, China and the UK examined the effects of the physical and psychosocial work environment on offshore workers’ health status [5–8]. Although Ross [7] reported good general health of offshore populations in the North Sea, some major health concerns were: sleeping problems, gastrointestinal and cardiovascular diseases.

**Table 1:** The Dutch offshore environment

Offshore operations are carried out on remote platforms in hazardous marine and industrial environments. Offshore workers work 12 hours a day for fourteen consecutive days, followed by fourteen days off work. The physical properties of the platforms (e.g. noise and motion levels) and the social factors of the job (e.g., being away from home) add to the uniqueness of the offshore work environment. In the Dutch offshore environment, it is still common to retire about 10 years before the official retirement age. A possible reason is that most offshore workers execute highly demanding physical work, which poses additional risks on workers’ safety and health making them more prone to retire early.	

Among older offshore workers, hypertension, diabetes, obesity and hypercholesterolemia were found to be the most frequently occurring disorders [9]. Moreover, older offshore workers have a greater restitution need for undisturbed sleep and are more susceptible to sleep disturbances from cabin noise or night-shift work activities [2] and need to recover from poor sleep quality during offshore shifts during leave periods [10]. By approaching HA@W from a life course perspective, this study investigates health dynamics among age groups to promote age-specific intervention program objectives.

Workplace interventions have been difficult to implement, reflected by disappointing transfer rates ranging between 10 and 50 % [11]. Several concepts, such as the intention-behaviour gap [12] or motivation to transfer [13] have been mentioned as explanations for the implementation problem. Other explanations concern both a lack of focus on the work context and integrated approaches. Despite these implementation issues, workplaces have specific features that make them a promising place for HA@W programs, such as peer/colleague support [11].

Intervention mapping (IM) has been shown to reduce implementation problems by considering specific environmental and population characteristics. Additionally, IM has been acknowledged by researchers as an important preparatory step towards developing health promotion programs [14]. IM provides a systematic framework for planning, development and implementation of evidence-based health promotion and prevention programs [15]. IM determines the discrepancies between current and desired conditions and involves six steps: (1) a needs assessment; (2) defining suitable program objectives; (3) theory-based intervention methods and practical applications; (4) an intervention program; (5) adoption, implementation and an (6) evaluation of the intervention. Moreover, IM employs a mixed method design, using qualitative and quantitative data. Although IM has been shown to be beneficial for the development of successful interventions, IM is hardly used due to high costs and time pressure [15]. In this study, the first two steps of IM were conducted. In step 1, needs assessments among offshore workers and their supervisors are performed and age-related differences in health status and work-related factors are investigated. In step 2, suitable program objectives for a future HA@W program in the offshore working population are defined.

## Method

This study employs a mixed-method design. A grounded theory-lite approach, was used to identify codes, concepts and categories (needs/contents) underlying HA@W offshore [16]. In the qualitative study, data were gathered in semi-structured interviews with supervisors and focus-groups among offshore workers (inductive process) to derive information on the needs/contents of a HA@W program (deductive process). The semi-structured interviews were used to perform an ethnography of the offshore population and to identify the management views on the needs/contents of a HA@W offshore program. Focus groups were used to perform a content analysis of the workers needs of a HA@W program offshore. Both interviews and focus-groups were conducted during working time offshore. Based on the results of the qualitative study, a HA@W questionnaire was developed for the subsequent quantitative study among offshore workers. Participation in this study was voluntarily and written informed consent was obtained for the qualitative and quantitative study. The study was announced by invitation emails, posters and mouth-to-mouth promotion. No exclusion criteria were defined. Semi-structured interviews and focus groups were conducted by the PI, who had elaborate training in interviewing and moderating skills. Notably, the PI had no personal relationships with any of the participants included in this study. Ethical approval was granted from the Medical Ethics Committee (reference number: M12.125779) of the University Medical Center Groningen (The Netherlands).

### Qualitative study

#### Participants and procedures

Twelve semi-structured interviews among offshore supervisors were conducted from October to November 2012. Purpose sampling was used to identify interviewees, representing the following departments: Human Resources; Health Safety and Environment; Health; Offshore Management and Operations. All invited interviewees took part in the study. Seven additional semi-structured interviews were held with five offshore installation managers and two offshore workers of the visited platforms, asking the same questions. All one-hour interviews were conducted in Dutch and were taped with two recording devices. The questions for the semi-structured interviews were developed in collaboration with members of the University’s HA@W research group and were piloted. Data were collected on socio-demographics, work environment characteristics and needs regarding future HA@W programs. Saturation was reached after completion of twelve interviews with offshore supervisors.

Six focus-groups with 6–10 offshore worker volunteers were conducted on five platforms in the Dutch Continental Shelf over a 3-week period in November 2012. Focus-groups were held in English or Dutch and bilingual interview protocols were prepared listing all questions and procedures to ensure comparability of the sessions. Per platform one or two focus-groups were conducted. All sessions were recorded. Questions covered five areas: the definition of healthy ageing, conditional requirements to stay mentally and physically healthy offshore until retirement; opinions about existing/former company health programs; ideas for future HA@W programs and identification of facilitating factors.

#### Analyses

All audio recordings were transcribed and anonymized by an independent research assistant. All authors analysed the transcripts and coded the content separately. Codes, concepts, categories and theories underlying HA@W offshore were identified. The results were discussed by all authors and summarized in a document listing the semantic categories and content components.

### Quantitative study

#### Participants and procedures

All offshore workers working on Dutch offshore platforms operated by the participating company, in the time period February 12, 2013 till March 1, 2013, were invited to participate in the study. No exclusion criteria were applied. Dutch and English versions of the questionnaire were handed out at check-in at the airport. Offshore workers completed the questionnaire during waiting time and returned the questionnaire before boarding the aircraft. Offshore workers who did not complete the questionnaire before the flight were allowed to take the questionnaire to the offshore platform and to hand the completed questionnaire to the Offshore Installation Manager who returned it to the researcher. Offshore workers were asked to complete the questions related to their last offshore stay.

#### Measurements

We considered a Cronbachs alpha of ≥ .8 to be an indicator of a good internal consistency [17].

#### Socio-demographics, health behaviour, job characteristics and food

Age, gender, height, weight, socioeconomic status, family situation, smoking (yes/no; packs) and drinking behaviour (yes/no; glasses), job title, shift work (yes/no), tenure (in years) and frequency of day tripping were assessed. Self-reported height and weight were used to calculate the body mass index (BMI) with BMI ≤ 18.5 = underweight, BMI 18.5–24.9 = normal weight, BMI ≥ 25 = overweight and BMI ≥ 30 = obese [18]. Five self-constructed items were used to assess food quality perceptions and eating/dieting behaviours (*α* = .85).

#### Ageing and human resource

Ageing aspects related to working conditions were assessed with five items [19]. Additionally, three self-constructed items on human resource aspects (*α* = .82) and one item concerning age discrimination were measured. All self-constructed items can be found in the Additional file 1.

#### Work ability and work functioning

Work ability was assessed with the overall single item (current work ability compared to lifetime best) from the Work Ability Index. [20] Health-related work functioning was measured with two self-constructed items: 1) work functioning on a ten-point Likert scale with 1 being the lowest work functioning to 10 being the highest work functioning and 2) satisfaction with work functioning on a five-point Likert scale, ranging from very satisfied to very dissatisfied.

#### Offshore environment

The physical offshore environment (e.g. living accommodations, workplaces) was measured with eight self-constructed items on a five-point Likert scale, ranging from very good to very bad (*α* = .83). Satisfaction with environmental stressors (e.g.: ventilation and noise) was measured with eight self-constructed items on a five-point Likert scale ranging from very satisfied to very unsatisfied (*α* = .87). The social environment offshore was measured with three self-constructed items about social atmosphere and relationships with colleagues (*α* = .80). Four items of the Dutch National Survey on Work Conditions 2011 were used to investigate relationships between colleagues [21]. Privacy aspects were assessed with two self-constructed items about satisfaction with sleep accommodations and privacy offshore on a five-point Likert scale ranging from very satisfied to very dissatisfied.

#### Health status and sickness absence

General health was measured with the reliable and valid Short Form-12 (SF-12) questionnaire [22]. The SF-12 measures health by physical and mental component summary scores, ranging from 0–100, with higher scores indicating better health [23]. Dutch norm cut-off scores were set at 51 [24]. In addition, the single-item score of the SF-12: In general would you say your health is: (5) excellent, (4) very good, (3) good, (2) fair or (1) poor?’ was used [25]. Chronic health conditions were measured with one item from the Dutch National Survey on Work Conditions 2011 [21].

#### Chronotype

Chronotype was determined with the validated Munich Chronotype Questionnaire [26]. Offshore workers were asked to complete the questions for two scenarios: working offshore and being at home. Chronotype was defined as the midsleep point (the half-way point between sleep onset and sleep end) when at home [27].

#### Need for recovery and dips

Need for Recovery was measured with a subscale of the Dutch questionnaire on Perception and Judgment of Work [28]. The need for recovery consists of eleven dichotomous items (yes/no) assessing short-term effects of a day of work. Total scores range from 0 to 100, with higher scores indicating higher need for recovery. A cut-off score of >36 was used to indicate increased need for recovery [29]. The reliability and validity of the need for recovery are good (α = 0.87) [28]. Physical and/or mental ‘dips’ during the two-week offshore work period were assessed with two self-constructed items.

#### Fatigue

Fatigue was measured with the eight-item ‘subjective experience of fatigue’ subscale of the Checklist Individual Strength (CIS-8) [30]. The Checklist Individual Strength is an appropriate instrument for measuring fatigue in the working population with a good reliability (α = 0.80–0.96) [30–32]. A seven-point Likert scale (1 = Yes, that is true to 7 = No that is not true) was used, with higher scores indicating prolonged fatigue. A cut score of ≥ 35 was used to indicate prolonged fatigue [33].

#### Work family conflict

Work Family Conflict was measured with one item of the Copenhagen Psychosocial Questionnaire II (COPSOQ-II): ‘Do you often feel a conflict between your work and your private life, making you want to be in both places at the same time?’[7]. Answering options ranged from 1 = Yes, often, to 4 = No, never; reliability and validity of the COPSOQ-II are good (α = 0.80) [7].

#### Analysis

Descriptive analyses were conducted for all variables. Age was categorized into three age groups according to offshore age distributions and company specific retirement regulations: < 45 (N = 127); 45–54 (*N* = 81) and ≥ 55 (*N* = 47). Subgroup analyses using univariate analyses (ANOVA) with post hoc Bonferroni adjustments were performed to investigate age differences. Offshore workers, who filled in less than 50 % of the questionnaire (*N* = 2) and who did not sign the informed consent (*N* = 10) were excluded from the analyses. We excluded offshore workers from the chronotype analysis if inconsistencies and nonconformities (interchange of 12-h with 24-h time scale) in the questionnaire were found. All analyses were conducted using SPSS version 21.

## Results

### Interviews and focus-groups

Twelve offshore supervisors *[s],* five Offshore Installation Managers *[o]* and two offshore workers *[wSSI]* were interviewed [for interviewee characteristics see Additional file 2] and 49 offshore workers *[wFG]* participated in six focus-groups [for focus-group participant characteristics see Additional file 3].

#### Characteristics of the offshore population

Interviewees characterized the offshore population as a male-dominated, knowledgeable, experienced and motivated group with a strong work mentality. Offshore workers noted that people working offshore have to adjust to offshore job prerequisites (e.g., working on remote locations) and have to possess certain social skills (e.g., being extraverted). Interviewees found it difficult to describe the certain social skills in more detail. It was stated that, people who choose this career path are usually very happy in their job. ‘*It is a profession that has to suit you, but once you are accustomed to it you never want to do other work’ [wSSI].* Offshore workers live and work together and it was mentioned that special group dynamics are formed in which offshore workers influence each other. Social ties tend to be very strong ‘*’If someone cannot perform a certain task, the task will be picked up by someone else in the group without turning a hair.’[s].*

#### Characteristics of offshore work

Positive aspects of offshore work included: financial benefits, free time, flexibility of living conditions, variation in work, adventurous work conditions and contact with colleagues. Being far away from home was mentioned as the main negative aspect of offshore work *‘The only thing I don’t like is that when something happens at home you are far away from family and friends.’[wFG],* along with logistic problems regarding work preparation and communication with the onshore office. Further, offshore workers mentioned that at times it felt like being in a ‘*golden cage’*, far away from home. ‘*If we ask people to work onshore they reply: I rather not because it would lower my income. Thus, we have created workers who have a golden chain around their ankles; tied to the offshore setting.’[s].*

#### Healthy ageing @ work offshore

Supervisors described the offshore population as an ageing workforce, with an age gap between 30 and 40 years. Offshore workers [wFG] noted that other factors related to offshore work, such as the ergonomic platform conditions, influenced ageing symptoms. First, wearing down of joints was mentioned due to climbing several flights of stairs a day and working in difficult positions. Second, organizational factors, like limited flexible work arrangements, were noted to likely influence health and social life offshore. ‘*Being 100 % fit and alert for 14 days gets increasingly difficult’* [wFG]. Moreover, an increased need for recovery on a daily base and accumulated exhaustion at the end of a fourteen day shift were reported.

Offshore workers spend nearly half a year offshore and it was identified as having ‘*two lives’ [wFG]*. Difficulties related to work family balance were stated. Offshore workers experienced interrole conflicts and work family conflict due to prolonged absences from home*.* An increased occurrence of work family conflict with increasing age has been noted by some offshore workers ‘A*s long as you have a family at home with small children, everything is all right. But once the children leave the house, wives start to nag a bit that we are away a lot.’[wFG].* Many offshore workers stated that the importance of having contact with family members and friends increased with age.

Offshore workers noted that HA@W was possible when they remain having fun at work and have varied work tasks. As prerequisite for HA@W offshore workers mentioned a good physical (work) environment, good working material and ergonomic adjustments at the workplace to ensure safety and health. Furthermore, privacy was mentioned by several offshore workers. *‘For me personally, privacy is becoming more important as I grow older.’[wFG]; ‘In the past few years I find it more annoying to share a cabin with somebody else.’[wFG]* The increasing desire for private accommodations by older offshore workers was explained by various age-related factors, such as embarrassment for frequent voiding at night and (invasive) drug administration.

#### Health and health behaviour

Supervisors noted that sickness absence rates among offshore workers were very low but that these rates did not reflect the true health status of offshore workers. The health mentality is shaped by offshore workers ‘macho-identity’ *‘No whining is accepted!’[s];’(…) many health complaints are camouflaged*’ *[s]* ‘*You do not get sick; I would never call in sick.’ [wFG]* and organizational components, such as salary: *‘It is more profitable to recuperate offshore’ [o]. S*everal health-related population characteristics, such as increased occurrences of chronic disease, high BMI’s, and decreased condition and fitness levels were noted by offshore workers. Offshore workers also mentioned safety concerns such as, being alone on a normally unmanned installation and being in a helicopter with an overweight/obese person. ‘*Last time I flew home, I was sitting between two people who had stretched their seatbelts to the maximum and still had to hold their breath. If anything would have happened it would have been impossible for me to escape. ‘[wSSI]* One quote summarized the nature of offshore work: ‘*You lead a Spartan life: Working, Eating and Sleeping.’[wFG].*

Food and nutrition were identified as major health concerns. Interviewees criticized the easy access of unhealthy food and the unhealthy eating behaviours of offshore workers. ‘*The problem remains the food offshore.’[s]; ‘Two warm meals a day is the rule rather than the exception.’ [o]; ‘You work from meal to meal, from coffee to coffee because it’s the only thing.’ [wFG].* Meals were seen as social gatherings, i.e., as one of the few pleasurable things offshore and as a compensation for many things offshore workers miss offshore.

Fatigue and its effects on safety, alertness and well-being were mentioned by offshore workers. The length of shifts was discussed by several interviewees and most agreed that fourteen days offshore might be too long from a health perspective. Further, a ‘dip’ was noted by several offshore workers, described as a day when they felt mentally and/or physically exhausted from the previous working days and their mental and cognitive capacities declined. ‘*On the tenth day there is a ‘dip’ and fatigue hits you’ [wSSI]*; ‘M*any have reached their limit after ten days, although they have four more days to go’ [wFG].* All offshore workers were concerned that this dip influenced safety offshore.

Sleep disruption due to environmental stressors (e.g. motion and noise of the platform); ergonomic requirements (e.g. length, quality of mattresses) and roommates (e.g. snoring) was noted. *‘In temporary living quarters there is much noise and you sleep restless. You wake up a few times a night, for example when the engine starts running again’ [wFG];*

Smoking was a controversial topic among the offshore workers. There were strong supporters and opponents of smoking offshore. The opponents requested a general smoking ban offshore, whereas the supporters made statements such as: *‘If a smoking ban was to be introduced offshore, I would look for a new job.’[wFG].*

#### Future HA@W programs

All interviewees noted that future HA@W programs should be offered voluntarily, include an element of fun and should consider the permanent lack of space offshore. The importance of initiators, role models and motivated people within the target group was mentioned *‘You need one or two initiators on a platform that can motivate people. For an outsider it is much harder to get through to these workers.’[s]. A*ny future program should be tailored to the needs of the offshore population and should be communicated effectively. *‘Everything comes down to: communication’ [s].* In the past, communication tools were not used appropriately resulting in information overload. *‘One is bombarded with emails, digital newsletters and more of those things.’ [wSSI].* Continuity and long-term commitment to future programs and interventions by the company were mentioned as major factors for future successes. *‘People are tired of ‘the flavour of the month’ or something that is a hot topic this year, but is forgotten the next.’ [o].* A participatory approach was advocated by offshore workers to raise awareness in an active fashion. ‘*When you set up a* HA@W *program, it is important to keep it vivid instead of being just another piece of paper.’ [wFG].*

The two main topics mentioned by offshore workers in terms of HA@W interventions were good food and good sleep. Offshore workers wanted a tailored food program offering healthy choices, better catering and food displays and individual coaching. Extra training on healthy cooking skills for chefs was suggested as well as mandatory fruit displays (healthy snacks) and a priori food choice to improve food quality.

### HA@work questionnaire

#### Sample characteristics

A total of *N* = 272 (61 %) offshore workers returned the questionnaire of which *N* = 260 (58 %) had complete data suitable for data analysis (Table 2). The majority of offshore workers worked day shifts (68 %) and did not have to take daytrips to other platforms (75 %). Overall, 38 % reported that they experience work family conflict regularly or often. Pearson correlations between the main continuous variables are shown in Table 3. For age, the highest correlation coefficient was found with BMI (*r* = .34, *N* = 249, *p* < .01). Correlation coefficients varied between−.02 and−.48. Although the correlation coefficients were generally low they provided an indication of the strengths and directions of the relationships.

**Table 2:** Characteristics of offshore workers

	Total	<45 years	45–54 years	≥55 years	Difference
	*N* = 260	*N* = 127 (50 %)	*N* = 81 (32 %)	*N* = 47 (18 %)	*p*-values
Age, years mean (sd)	44.14 (10.7)	35.14 (6.7)^ab^	49.89 (2.6)^ac^	58.55 (2.5)^bc^	.000
Function, years mean (sd)	11.26 (10.2)	7.05 (6.5)^ab^	13.22 (10.3)^ac^	19.83 (11.9)^bc^	.000
Work Offshore, years mean (sd)	11.3 (9.8)	5.7 (5.2)^ab^	14.02 (9.1)^ac^	22.3 (9.5)^bc^	.000
Number of children, *N* (%)	1.69 (1.3)	1.24 (1.2)^ab^	2.09 (1.2)^a^	2.23 (1.1)^b^	.000
BMI, mean (sd)	27.01 (3.7)	26.29 (3.7)^b^	27.36 (3.3)	28.6 (3.6)^b^	.001
Gender (Male) *N* (%)	251 (97.3)	120 (95.2)	80 (98.8)	47 (100)	.141
Education, *N* (%)					.054
Low	63 (24.5)	27 (21.4)	24 (30.0)	12 (25.5)	−
Middle	137 (53.3)	73 (57.9)	39 (48.8)	23 (48.9)	−
High	53 (20.6)	26 (20.6)	13 (16.3)	12 (25.5)	−
Other	4 (1.6)	−	−	−	−
Divorced (Yes)	56 (21.7)	14 (11.1)^ab^	26 (32.1)^a^	15 (31.9)^b^	.000
Family situation, *N* (%)					.005
Married without kids at home	74 (28.7)	32 (25.4)	17 (21.0)	25 (53.2)	−
Married with kids at home	129 (50)	63 (50)	44 (54.3)	18 (38.3)	−
Single parent	13 (5)	5 (4.0)	6 (7.4)	2 (4.3)	−
Single	32 (12.4)	21 (16.7)	9 (11.1)	2 (4.3)	−
Other	10 (3.9)	5 (4)	5 (6.2)	−	−
WFC^*^, mean (sd)	1.73 (.6)	1.78 (.6)	1.70 (.5)	1.64 (.5)	.308
Shift work, *N* (%)		^a^	^a^		.035
No	176 (68.2)	76 (60.8)	60 (74.1)	37 (78.7)	−
Yes, regularly	54 (20.9)	35 (28)	10 (12.3)	7 (14.9)	−
Yes, sometimes	28 (10.9)	14 (11.2)	11 (13.6)	3 (6.4)	−
Day tripper, *N* (%)					.646
No	190 (74.5)	90 (73.2)	58 (72.5)	40 (85.1)	−
Yes, regularly	30 (11.8)	14 (11.4)	11 (13.8)	3 (6.4)	−
Yes, sometimes	34 (13.3)	18 (14.6)	11 (13.8)	4 (8.5)	−

Participants were excluded from the analysis if they had missing data

^*^WFC (Work-family conflict)

^a^Significant difference between group < 45 years with group 45–54 years

^b^Significant difference between group < 45 years with group ≥ 55 years

^c^Significant difference between group 45–54 years with group ≥ 55 years

**Table 3:** Pearson correlations between age, need for recovery, subjective fatigue, BMI, subjective well-being, and work ability

	Age	NFR	CIS	BMI	SWB	WAI	N
Age	____						255
NFR	−.02	____					246
CIS-8	.09	−.33**	____				244
BMI	.34**	−.12	.09	____			249
SWB	.22**	.22**	−.11	.11	____		253
WAI	−.17**	−.17**	.15*	−.12	−.48**	____	248

*NFR* Need for recovery, *CIS* Checklist Individual Strength, *BMI* Body mass index, *SWB* Subjective well-being. *WAI* Work ability index, Statistical significance at *p < .05 and **p < .01

#### Health status, health behaviours and sickness absence

Physical and mental component scores (SF-12) were above the Dutch norm for the overall sample and the age subgroups (Table 4). Two thirds of offshore workers (67 %) reported a chronic health condition; most commonly reported were musculoskeletal (*N* = 20) and cardiovascular (*N* = 13) disorders. [For an overview of participants reported chronic conditions see Additional file 4]. Of those offshore workers reporting a chronic health condition, 58 % indicated that they can perform their work without any health complaints and 44 % indicated that they had to adjust their work or had to work slower. When asked about the likelihood of pursuing their current job in the next two years, all offshore workers indicated a more than 90 % likelihood of staying in their current position. The mean BMI score of offshore workers was 27 (SD = 3.7); 46 % were overweight and 21 % were obese. A significant difference in BMI was found between offshore workers aged < 45 and ≥ 55 years (mean 26.3 vs. 28.6; *p* = .001). Food offshore was scored as bad or really bad (75 %). Sixty-four percent indicated that they consumed at least two warm meals a day offshore. Overall, sickness absence was low (Table 4).

**Table 4:** General health, health behaviours and sickness absence

	Total	<45 years	45–54 years	≥55 years	Difference *p*-values
SF-12^*^ single item, *N* (%)		^ab^	^a^	^b^	.005
Excellent	40 (15.6)	21 (16.7)	13 (16.3)	6 (12.8)	−
Very Good	92 (35.8)	60 (47.6)	17 (21.3)	13 (40.4)	−
Good	120 (46.7)	45 (35.7)	49 (61.3)	25 (53.2)	−
Fair	5 (1.9)	−	1 (1.3)	3 (6.4)	−
SF-12 MCS, mean (sd)	54.48 (5.66)	54.1 (5.75)	54.36 (5.44)	55.74 (5.44)	.367
SF-12 PCS, mean (sd)	52.91 (4.74)	53.85 (3.6)^a^	52.63 (4.24)	51.23 (7.06)^a^	.013
Sickness absence, *N* (%)					.630
0 Days	187 (73.3)	90 (71.4)	60 (75.9)	35 (76.1)	−
1–9 Days	54 (21.2)	29 (23.0)	13 (16.5)	10 (21.7)	−
10–24 Days	6 (2.4)	2 (1.6)	4 (5.1)	1 (2.2)	−
25–99 Days	7 (2.7)	4 (3.2)	2 (2.5)	−	−
100–365 Days	1 (0.4)	1 (0.8)	−	−	−
Smoking (Yes), *N* (%)	100 (38.6)	57 (55.1)	26 (32.1)	15 (31.9)	.108
Packs, mean (sd)	3.04 (1.9)	3.23 (2)	2.73 (1.8)	2.85 (2)	.526
Alcohol (Yes), *N* (%)	217 (84.1)	110 (86.6)	63 (77.8)	41 (89.1)	.141
Glasses, mean (sd)	7.13 (5.7)	6.29 (5.1)	7.79 (6.2)	8.25 (6.4)	.103

^*^SF-12 scores of the Dutch version of the questionnaires

^a^Significant difference between group < 45 years with group 45–54 years

^b^Significant difference between group < 45 years with group ≥ 55 years

#### Fatigue, dips, need for recovery, chronotype and sleeping accommodations

**Fig. 1:**
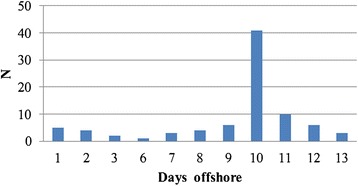
Dip days. This figure shows the graphical distribution of experienced dip days during the fourteen-day shifts of offshore workers

In total, 181 offshore workers (73 %) reported prolonged fatigue (Table 5). Across the age groups, small differences in fatigue scores were observed (M _< 45_ = 36.0; M _45–54_ = 36.4; M _≥ 55_ = 37.6). Overall, 41 % reported that they experienced a dip at some point during their shift offshore. Dips were mainly experienced at day 10 or 11 (60 %) (Fig. 1), 43 % experienced the dip both physically and mentally, whereas 39 % only mentally (Table 5).

**Table 5:** Fatigue, Dips, Need for Recovery and Chronotype

	Total	<45 yrs	45–54 yrs	≥55 yrs	Difference *p*-values
CIS-8^*^, mean (sd)	36.36 (4.24)	35.95 (4.07)	36.36 (4.41)	37.58 (4.28)	.085
Dip days (Yes) (*N*, %)	96 (41)	53 (55)	25 (26)	16 (17)	.358
Dip experience (*N*, %)					.685
Mentally	41 (39)	24 (41)	10 (33)	7 (44)	−
Physically	19 (18)	11 (19)	5 (17)	1 (6)	−
Both	46 (43)	23 (40)	15 (50)	8 (50)	−
NFR°, (scale 0–100)					
Median IQR	20 (9.09–36.36)	18.18	27.27	18.18	−
Mean (sd)	26.42 (18.28)	26.57 (18.75)	26.41 (18.25)	24.37 (16.73)	.772
Midsleep duration, mean (sd)					
Offshore	2.72 (2.14)	2.93 (2.38)	2.55 (1.86)	2.24 (0.53)	.156
Free Days	3.75 (0.96)	3.97 (1.04)	3.53 (0.93)	3.63 (0.78)	.348
Sleep duration, mean (sd)					
Offshore	7.18 (0.99)	7.21 (1.06)	7.15 (0.94)	7.03 (0.78)	.605
Free Days	7.82 (1.01)	7.88 (1.06)	7.78 (1.02)	7.74 (0.88)	.235

Due to missing values the sum scores are not equal to the total; ^*^CIS = checklist individual strength; °NFR = need for recovery scores

The median need for recovery score of the total sample was 20 (IQR = 9.09 to 36.36) and no differences were found between age groups. One-third indicated a high level of need for recovery. For midsleep and sleep duration, differences were found between age groups and within settings (offshore vs free days) (see Table 5). Older offshore workers reported shorter sleep durations and earlier chronotypes. The majority (74 %) of the offshore workers rated their sleeping accommodation as good or very good. Offshore workers were (very) satisfied with the sleeping accommodation and privacy offshore (68 and 61 %, respectively).

#### Work ability, work functioning and ageing at work

Offshore workers reported a high mean work ability of M = 8.41 (SD = 1.06). When looking at age and work ability, significant differences were found between offshore workers aged < 45 and 45–54 years (mean 8.63 vs. 8.19; *p* = .005) and offshore workers aged <45 and >55 years (mean 8.63 vs. 8.22; *p* = .028) (Table 6).

**Table 6:** Work Ability and Work Functioning

	Total	<45 yrs	45–54 yrs	≥55 yrs	Difference *p*-values
Work Ability Index, mean (SD)					
overall-item (0–10)	8.41 (1.06)	8.63 (0.92)^ab^	8.19 (1.09)^a^	8.22 (1.15)^b^	.008
physical demands (0–5)	4.3 (0.54)	4.37 (0.52)	4.21 (0.47)	4.26 (0.61)	.078
mental demands (0–5)	4.22 (0.56)	4.25 (0.54)	4.24 (0.59)	4.22 (0.47)	.940
WF^*^, mean (SD) (range 1–10)	8.20 (0.9)	8.33 (0.83)	8.09 (0.82)	8.09 (0.96)	.091
WF_satisfaction, *N* (%)					.409
Very satisfied	62 (24.3)	29 (23.2)	16 (20.3)	17 (37)	−
Satisfied	168 (65.9)	84 (67.2)	56 (70.9)	25 (54.3)	−
Neutral	15 (5.9)	7 (5.6)	3 (3.8)	4 (8.7)	−
Dissatisfied	3 (1.2)	1 (0.8)	1 (1.3)	−	−
Very dissatisfied	7 (2.7)	4 (3.2)	3 (3.8)	−	−

^*^WF (Work functioning)

^a^Significant difference between group < 45 years with group 45–54 years

^b^Significant difference between group < 45 years with group ≥ 55 years

Work functioning of offshore workers was good. More than 90 % of the total sample indicated that they were either satisfied or very satisfied with their work functioning (Table 6). Overall, 11 % of offshore workers reported problems in working life due to ageing and 11 % experienced barriers in performing their work tasks due to ageing. Other ageing-related problems concerned sleep, concentration and adaptation to shift work. The mean planned retirement age was 63.61 years (SD = 4.04). A third of the offshore workers mentioned that they can perform many work tasks better today compared to ten years ago, e.g., they reported a higher efficiency due to faster decision making and an improved ability to plan ahead and to analyse situations more accurately as well as better mentoring/supervising skills. The overall quality of the physical offshore environment was rated good, but ventilation and noise levels were rated poorly. The social atmosphere offshore was perceived as good. The majority of offshore workers (93 %) reported good or very good relationships with colleagues.

## Discussion

In the present study, a mixed-method design was used for the needs assessment (IM step 1) to define objectives for future HA@W programs offshore (IM step 2). Age-related differences regarding health and work-related factors in the Dutch Continental Shelf were investigated. Three main objectives for future HA@W programs were identified: work, food and sleep.

### Health status, health behaviours and sickness absence

In line with previous studies [7], the self-reported health status of offshore workers was good and scores for physical (PCS) and mental (MCS) functioning were above the cut-off point for the Dutch population. A significant decrease in physical component summary scores and a (non-significant) increase in mental component summary scores with age were observed, as shown in previous studies [34], The value of food offshore is overarching. The semi-structured interviews and focus-groups revealed that offshore workers perceive meal times as extremely pleasurable, which was confirmed by the questionnaire results. A similar observation was made by Parkes in 2003 who examined a British offshore population. She states in her article that meal times ‘provide the main focus for relaxation and social interactions’[35]. The majority of the offshore workers indicated that they consume at least two warm meals a day and that working offshore affects their weight negatively (BMI: M = 27, SD = 3.7, range 18.8–40.6). An earlier study on Norwegian offshore workers found similar mean BMI scores of 26 (range 19–37) [36]. In the present study, 67 % of the offshore workers are overweight, of which 21 % are obese. In contrast, Parkes et al. [37] found, that 47 % of British offshore workers were overweight and 8 % were obese [37]. Offshore workers in our study are considerably heavier, with more obese and overweight offshore workers compared to British offshore workers. Differences could be due to different cultures, work conditions or sample characteristics. Future comparative research should further investigate these differences. Although sickness absences were low, it is important to note, that offshore workers with minor ailments remain on offshore platforms to recuperate (either in the sickbay or in their cabin) and those days are counted as being at work rather than off sick.

### Fatigue, dips, need for recovery and chronotype and sleeping accommodations

In line with findings from other offshore studies, sleep problems were identified as one of the major health concerns of offshore workers even though the majority of our sample (68 %) worked only day shifts [5, 7]. Although severe fatigue was reported across all age groups, older offshore workers showed slightly higher fatigue scores compared to the youngest offshore workers. Severe fatigue is associated with sick leave and work disability and can pose potential threats to health and safety [31]. In addition, 41 % of the offshore workers indicated that they experience a dip at some point during their offshore shift. These dips were mainly experienced at day 10 or 11 (60 %). The combination of severe fatigue and dip experiences is potentially dangerous and harmful to offshore workers health and safety and should be addressed in future HA@W programs. To our knowledge, the phenomenon of dips among offshore workers has not been previously identified and more research is needed to further examine this phenomenon.

Need for recovery was not significantly related to age, which is consistent with a recent study on seafarers [29]. Offshore workers reported earlier chronotypes compared to the chronotypes of a Dutch comparison study [27]. With increasing age, offshore workers reported shorter sleep durations and earlier chronotypes. This age-dependency of chronotype has earlier been demonstrated by Roenneberg [38].

### Work ability, work functioning and ageing

Work ability and work functioning were high among all offshore workers. However, work ability index scores differed significantly between the youngest offshore workers and the older age groups, indicating a possible decrease in work ability with age. These findings are in line with other studies on work ability among auxiliary work-forces [39, 40] and are likely to be explained by general biological age-related health declines. Further, Bridger and Bennett [40] found that the interaction between BMI and age is the best predictor of work ability, e.g. increased BMI scores had deleterious effects on older seafarers work ability [40.] It is interesting to note, that 37 % of offshore workers in the oldest age category reported that they are very dissatisfied with their current work functioning. Further research is needed to explore the reasons for the dissatisfaction with work functioning.

### Strengths and limitations

A strength of this study is the use of a mixed methods approach. We have included views and information from different stakeholders and offshore workers (target population) and have gained important insights regarding the physical and psychosocial working conditions as well as the needs of offshore workers in the Dutch Continental Shelf in relation to HA@W. Limitations of the study mainly concern the use of self-constructed questionnaire items and the potential for recall bias. Self-constructed questionnaire items were used due to a lack of questionnaires targeted to the offshore work environment and a lack of validated tools to assess new constructs (e.g. dip day). Future studies should develop validated questionnaires for this population. Recall bias has to be considered when interpreting the results, because offshore workers were asked to indicate their perceptions and experiences with respect to the last time they were offshore (usually two weeks prior to questionnaire completion). It should be noted, that the results on several health variables might be biased. It could be that self-reported health is positively skewed due to the healthy worker effect and mandatory regular and stringent medical examinations. Furthermore, health outcomes might be underestimated due to the ‘macho-culture’ on platforms. Longitudinal data is needed to verify results and evaluate future HA@W programs offshore.

### Implications

Performing step 1 and 2 of IM helped to identify target areas and suitable program objectives for a future HA@W program offshore. Considering specific environmental and population characteristics offshore is likely to increase the transfer and success rates of future HA@W programs. For example, we were able to identify some unknown health concerns to the Dutch Continental Shelf offshore sector (i.e. high prevalence of ‘dip-days’ and severely fatigued offshore workers on day-shifts), which may have gone unnoticed. It is important that organizations act as facilitators to help offshore workers stay healthy across the lifespan, as they spend a significant amount of time away from home at the worksite. This study contributes to the overall knowledge on offshore populations and HA@W program objectives. The outcomes of the offshore needs assessment will guide the next step of the IM approach towards the development of a HA@W program. Future studies have to investigate possible causes of dip occurrences and high fatigue scores (IM step 3) to identify suitable interventions (IM step 4) for a HA@W program offshore (IM step 5) and to rigorously evaluate the program (IM step 6).

## Conclusions

The needs assessment (IM step 1) identified offshore workers characteristics and program objectives for future HA@W programs. Work, food and sleep aspects were recognized as suitable program objectives (IM step 2) in the offshore population. In the next step (IM step 3) theory based intervention methods will be selected to develop and evaluate a future HA@W program offshore (IM step 4–6).

## Additional files

Additional file 1: **Self-constructed questionnaire items.** This table lists all the self-constructed questions we asked offshore workers in the questionnaire. (PDF 166 kb)

Additional file 2: **Interview participant characteristics.** This table lists the interviewee characteristics. (PDF 16 kb)

Additional file 3: **Focus-group (FG) participant characteristics.** This table lists the focus-group participant characteristics. (PDF 157 kb)

Additional file 4: **Chronic diseases.** This table lists the amount of reported chronic health conditions of the sample. (PDF 168 kb)

## Abbreviations

*[o]*: offshore installation managers
*[s]*: offshore supervisors
*[wFG]*: offshore workers from focus-groups
*[wSSI]*: offshore workers from semi-structured interviews
BMI: body mass index
CIS-8: 8-item Checklist Individual Strength scores
COPSOQ-II: Copenhagen Psychosocial Questionnaire II
HA@W: healthy ageing at work
IM: intervention mapping
NAM: Nederlandse Aardolie Maatschappij B.V
SF-12: short form-12
