# Supplementary material for: Work, eat and sleep: towards a healthy ageing at work program offshore
Source: BMC Public Health. 2016 Feb 9;16:134. doi: 10.1186/s12889-016-2807-5 (PMC4748638; doi:10.1186/s12889-016-2807-5)
Supplement: Supplementary file 1 — Self-constructed questionnaire items. This table lists all the self-constructed questions we asked offshore workers in the questionnaire. (PDF 166 kb) [file 12889_2016_2807_MOESM1_ESM.pdf]

Additional file 1. Self-constructed questionnaire items

Constructs	Questions
Ageing & Human Resources	• At what age do you plan to retire?<br>• Would you work longer if you would have one of the following options: (Flexible work hours, job sharing, part-time work, other)<br>• With increasing age, it is important to me to: (Have more privacy, eat differently, have a good night's rest, stay in close contact with family and friends, other)<br>• Have you been discriminated against due to your age in the last 12month?
Work Functioning	• If you could give yourself a score, which score would you give yourself for your functioning at work?<br>• How satisfied are you with your functioning at work?
Physical Environment	• In general, how would you rate the following environments? (Sleeping accommodations, ALQs/TLQs, canteen, recreation rooms, exercise facilities, your workplace, heliport)
Environmental stressors	• Please indicate how satisfied you are with the following environmental factors. (ventilation, illumination, temperature, noise, air pollution, motion of the platform, smell, interior of accommodation)
Social Environment	• How would you rate the social atmosphere on-board?<br>• How would you characterize your working relationship with your colleagues?<br>• Do you feel accepted by permanent staff? (for contractors)
Privacy	• How satisfied are you with the (sleeping) accommodations?<br>• How satisfied are you with your privacy situation offshore?
Food Offshore	• In general, how would you rate the food, which is provided offshore?<br>• How do you rate the food choice/variability, which is provided offshore?<br>• How do you rate the food quality, which is provided offshore?

	• How do you rate the preparation of food?<br>• How often do you eat a warm meal during a regular working day?
Dips Offshore	• If you are offshore for 2 weeks, do you, at some point, experience a ‘dip’ (mentally and or physically)?<br>• During this ‘dip’ are you: mentally worn out; physically worn out or both mentally and physically worn out?
