# Supplementary material for: Work, eat and sleep: towards a healthy ageing at work program offshore
Source: BMC Public Health. 2016 Feb 9;16:134. doi: 10.1186/s12889-016-2807-5 (PMC4748638; doi:10.1186/s12889-016-2807-5)
Supplement: Supplementary file 2 — Interview participant characteristics. This table lists the interviewee characteristics. (PDF 16 kb) [file 12889_2016_2807_MOESM2_ESM.pdf]

**Additional file 2.** Interview participant characteristics

| | Gender | Age | Department | | Gender | Age | Department |
|---|---|---|---|---|---|---|---|
| Supervisors [s] | | | | OIMs [o] | | | |
| 1 | Male | 40 | Offshore | **13** | Male | 53 | Offshore |
| 2 | Female | 51 | Health | **14** | Male | 42 | Offshore |
| 3 | Female | 43 | HSE | **15** | Male | 59 | Offshore |
| 4 | Female | 35 | Health | **16** | Male | 36 | Offshore |
| 5 | Male | 58 | Health | **17** | Male | 56 | Offshore |
| 6 | Male | 54 | HR | | | | |
| 7 | Female | 43 | HSE | Workers [wSSI] | | | |
| 8 | Male | 48 | Operations | **18** | Male | n/a | Offshore |
| 9 | Male | 58 | HSE | **19** | Male | n/a | Offshore |
| 10 | Male | 53 | HSE | | | | |
| 11 | Male | 53 | Operations | | | | |
| 12 | Female | 27 | HR | | | | |

[a] HSE (Health Safety and Environment); HR (Human Resources)
