# Supplementary material for: Work, eat and sleep: towards a healthy ageing at work program offshore
Source: BMC Public Health. 2016 Feb 9;16:134. doi: 10.1186/s12889-016-2807-5 (PMC4748638; doi:10.1186/s12889-016-2807-5)
Supplement: Supplementary file 3 — Focus-group (FG) participant characteristics. This table lists the focus-group participant characteristics. (PDF 157 kb) [file 12889_2016_2807_MOESM3_ESM.pdf]

Additional file 3. Focus-group (FG) participant characteristics

**FG AWG**	**Age**	**Job**	**Company**	**FG K15–A**	**Age**	**Job**	**Company**
Worker 1	n/a	Fitter	Contractor	Worker 1	36	Foreman	Contractor
Worker 2	n/a	Fitter	Contractor	Worker 2	37	Electrician	Contractor
Worker 3	39	SOT	NAM	Worker 4	36	Fitter	Contractor
Worker 4	n/a	Fitter	Contractor	Worker 5	54	Operations	NAM
Worker 5	n/a	Mechanical	Contractor	Worker 6	52	Process Operator	NAM
Worker 6	n/a	Process Safety	NAM	Worker 7	52	n/a	Contractor
Worker 7	32	Operator, SOT	NAM	Worker 8	27	n/a	Contractor
Worker 8	33	Operator	NAM	Worker 9	48	Supervisor Maintenance	NAM
Worker 9	57	Crane mechanic	NAM				
**FG L2**				**FG K14–C**			
Worker 1	32	Foreman	n/a	Worker 1	48	Assistant Platform Supervisor	NAM
Worker 2	n/a	Isolation mechanic	n/a	Worker 2	32	Offshore Setter	Contractor
Worker 3	n/a	Fitter	Contractor	Worker 3	33	CET Supervisor	NAM
Worker 4	41	Foreman	Contractor	Worker 4	45	Project supervisor	Contractor
Worker 5	41	Scaffolder	n/a	Worker 5	28	Scaffolder	Contractor
Worker 6	n/a	Scaffolder	n/a	Worker 6	41	SOT	NAM
**FG L13 (1)**				**FG L13 (2)**			
Worker 1	39	Welder	Contractor	Worker 1	42	Construction Painter	Contractor
Worker 2	34	Fire protection	Contractor	Worker 2	42	Construction Painter	Contractor
Worker 3	32	Steward	Contractor	Worker 3	38	Construction Painter	Contractor
Worker 4	56	Fire protection	Contractor	Worker 4	41	Construction Painter	Contractor
Worker 5	64	Supervisor	n/a	Worker 5	55	Sr Instrument. electrician	NAM
Worker 6	57	Operator	NAM	Worker 6	22	Construction Painter	Contractor
Worker 7	n/a	E&I Maint & Operator	Contractor	Worker 7	45	Painter	Contractor
Worker 8	44	Chemical Analyst	NAM				
Worker 9	47	Fitter	Contractor				
Worker 10	67	n/a	Contractor				
Worker 11	40	Fitter	Contractor				

[a] SOT (Senior Operation Technician); E&I Maint (Electricity and Instrumentation Maintenance)
