# Supplementary material for: Work, eat and sleep: towards a healthy ageing at work program offshore
Source: BMC Public Health. 2016 Feb 9;16:134. doi: 10.1186/s12889-016-2807-5 (PMC4748638; doi:10.1186/s12889-016-2807-5)
Supplement: Supplementary file 4 — Chronic diseases. This table lists the amount of reported chronic health conditions of the sample. (PDF 168 kb) [file 12889_2016_2807_MOESM4_ESM.pdf]

Additional file 4. Chronic diseases

	Total	< 45 years	45 – 54 years	≥ 55 years	Difference p-values
Psychiatric disorders	1	1	0	0	–
Diabetes	4	0[ab]	1[ac]	3[bc]	.010
Skin diseases	4	1	2	1	–
Migraine	6	3	2	1	–
Asthma	7	3	4	0	–
Hearing problems	8	3	4	1	–
Intestinal disorders	8	5	2	1	–
Vision problems	9	3	4	2	–
Cardiovascular disorders	13	1[b]	5	7[b]	.001
Other disorders	18	8	3[c]	7[c]	.049
Musculoskeletal disorders	20*	4[b]	6[c]	9[bc]	.001

[*] One participant had to be included for the subgroups analyses because we had no date of birth

a Significant difference between group < 45 years with group 45 – 54 years

[b] Significant difference between group < 45 years with group ≥ 55 years

[c] Significant difference between group 45 – 54 years with group ≥ 55 years
